# The Circulating GRP78/BiP Is a Marker of Metabolic Diseases and Atherosclerosis: Bringing Endoplasmic Reticulum Stress into the Clinical Scenario

**DOI:** 10.3390/jcm8111793

**Published:** 2019-10-26

**Authors:** Josefa Girona, Cèlia Rodríguez-Borjabad, Daiana Ibarretxe, Joan-Carles Vallvé, Raimon Ferré, Mercedes Heras, Ricardo Rodríguez-Calvo, Sandra Guaita-Esteruelas, Neus Martínez-Micaelo, Núria Plana, Lluís Masana

**Affiliations:** 1 Vascular Medicine and Metabolism Unit, Research Unit on Lipids and Atherosclerosis, Sant Joan University Hospital, Universitat Rovira i Virgili, IISPV, 43201 Reus, Spain; josefa.girona@urv.cat (J.G.); celia.nutricio@gmail.com (C.R.-B.); daiana.ibarretxe@urv.cat (D.I.); jc.vallve@urv.cat (J.-C.V.); rferre@grupsagessa.cat (R.F.); mercedes.heras@urv.cat (M.H.); ricardo.rodriguez@ciberdem.com (R.R.-C.); sandra.guaita@urv.cat (S.G.-E.); neus.martinez@urv.cat (N.M.-M.); nplana@grupsagessa.cat (N.P.); 2 Spanish Biomedical Research Centre in Diabetes and Associated Metabolic Disorders (CIBERDEM), 28029 Madrid, Spain

**Keywords:** GRP78/BiP, endoplasmic reticulum stress, atherosclerosis, carotid intima–media thickness, obesity, type 2 diabetes, metabolic syndrome, cardiovascular risk, fenofibrate/niacin treatment

## Abstract

Background: Glucose-regulated protein 78/Binding immunoglobulin protein (GRP78/BiP) is a protein associated with endoplasmic reticulum stress and is upregulated by metabolic alterations at the tissue-level, such as hypoxia or glucose deprivation, and it is hyper-expressed in fat tissue of obese individuals. Objective: To investigate the role of the GRP78/BiP level as a metabolic and vascular disease biomarker in patients with type 2 diabetes (DM), obesity and metabolic syndrome (MS). Methods: Four hundred and five patients were recruited, of whom 52.5% were obese, 72.8% had DM, and 78.6% had MS. The intimae media thickness (cIMT) was assessed by ultrasonography. The plasma GRP78/BiP concentration was determined, and its association with metabolic and vascular parameters was assessed. Circulating GRP78/BiP was also prospectively measured in 30 DM patients before and after fenofibrate/niacin treatment and 30 healthy controls. Results: In the cross-sectional study, the GRP78/BiP level was significantly higher in the patients with obesity, DM, and MS. Age-, gender- and BMI-adjusted GRP78/BiP was directly associated with LDL-cholesterol, non-HDL-cholesterol, triglycerides, apoB, and cIMT. GRP78/BiP was positively associated to carotid plaque presence in the adjusted model, irrespective of obesity, DM and MS. In the prospective study, nicotinic acid treatment produced a significant reduction in the GRP78/BiP levels that was not observed with fenofibrate. Conclusions: GRP78/BiP plasma concentrations are increased in patients with both metabolic derangements and subclinical atherosclerosis. GRP78/BiP could be a useful marker of metabolic and cardiovascular risk.

## 1. Introduction

Glucose-regulated protein 78/Binding immunoglobulin protein (GRP78/BiP) is an endoplasmic reticulum stress (ERS) protein that belongs to the Hsp70 multigene family and is located in the inner membrane of the endoplasmic reticulum. Its main function is associated with the unfolded protein response (UPR) in ERS situations. GRP78/BiP chaperons newly synthesized proteins until full maturation. In ERS situations, the proteins are titrated away, which frees ERS proteins that function to reduce protein synthesis and increase misfolded protein degradation and the protein-folding capacity [1]. GRP78/BiP is overexpressed in several tissues under ERS. The GRP78/BiP mRNA levels are elevated in the livers of obese mice, and high glucose levels result in reduced GRP78/BiP expression [2]. Additionally, the GRP78/BiP levels are increased in the adipose tissue of patients with diabetes and obesity [3,4]. Hypoxia and glucose deprivation account for the induction of GRP78/BiP [5,6]. Interestingly, this protein has also been detected in cell membranes, where it acts as a multireceptor and signal receptor transducer and mediates other functions [7,8]. These aspects are particularly relevant in some neoplastic tissues [9]. GRP78/BiP is released into culture medium from challenged cells to induce ERS. A soluble part of the protein can be detected in circulation, probably due to active secretion rather than simply a result of cell necrosis or apoptosis [10,11,12]. 

Alterations in ER homeostasis have been observed in obese and diabetic subjects [13]. The pathophysiological role of the UPR in obesity, insulin resistance and diabetes has been demonstrated in several studies in animal models [14] and humans [15,16]. One of the main findings was the demonstration that both genetically and diet-induced obese mice exhibited chronic activation of the UPR [2]. Consistent with these findings, treatment of obese and diabetic mice with chemical chaperones alleviated ERS and restored glucose homeostasis in the liver, muscle, and adipose tissues [17]. Furthermore, mice heterozygous for Grp78/BiP (*Grp78^+/−^*) were protected from the metabolic disorders linked to a high-fat diet [18]. A decrease in the BMI resulting from bariatric surgery reduced ERS in insulin-resistant, obese, human patients [19]. In this regard, physical exercise alleviates ERS in obese individuals through reduction of GRP78/BiP expression and release [4].

Atherosclerosis is the major cause of cardiovascular disease, and UPR activation occurs at all stages of atherosclerotic lesion development. GRP78/BiP has been found to be highly expressed in macrophages, smooth muscle cells, and endothelial cells of atherosclerotic lesions [20]. Increased ERS occurs in unstable plaques, suggesting that ERS-induced apoptosis of smooth muscle cells and macrophages may contribute to plaque vulnerability. Moreover, unstable atherosclerotic plaques present abnormal numbers of apoptotic cells, which is related to ERS [21] mainly via robust CHOP expression. ERS markers, such as GRP78/BiP, are strongly associated with atherosclerotic plaques in human coronary artery lesions [22]. Hemodynamic shear stress in atherosclerotic regions regulates GRP78/BiP expression in vivo and in vitro, and GRP78/BiP upregulation in the endothelium has been hypothesized to provide a protective compensatory effect in response to ERS within early or developing atherosclerotic lesions [23].

ERS is a pathophysiological process that is involved in many metabolic derangements. Therefore, an ERS biomarker should be highly informative at the clinical level. 

To the best of our knowledge, no studies have addressed the relationship between GRP78/BiP, diabetes, metabolic alterations, and subclinical atherosclerosis. In the present study, we have studied the associations between GRP78/BiP and metabolic indexes and atherosclerosis in patients with obesity, type 2 diabetes mellitus (DM), and/or metabolic syndrome (MS). We have also investigated the effect of lipid-modifying drugs on GRP78/BiP in patients with DM.

## 2. Research Design and Methods 

### 2.1. Design and Study Subjects

Cross-Sectional Study: For the cross-sectional study, we recruited 405 consecutive individuals attending the vascular medicine and metabolism unit of our university hospital due to lipid metabolism disturbances and associated disorders (obesity, DM, and MS) who were willing to participate. DM, MS, and obesity were diagnosed according to standard clinical criteria. Prediabetes was diagnosed according to the fasting glucose level (>100 mg/dL and <126 mg/dL). Subjects with chronic lung, renal, or liver disease, cancer, or any other serious disease were excluded. Patients on lipid-lowering drugs underwent a 6-week wash-out period (8 weeks if they were on fibrates). Anamnesis, anthropometric, and physical examination data were recorded. 

Prospective Study: GRP78/BiP was analyzed in deep-frozen stored sera from 29 patients with DM and 30 gender- and age-matched, apparently healthy, individuals (control group) who participated in an open randomized control trial to evaluate the impact of fenofibrate and niacin on HDL quality in DM patients. The details of this study including the flow chart scheme have been published [24] (ClinicalTrials.gov Identifier: NCT02153879). Briefly, after a 6-week lipid-lowering drug wash-out period, the patients with DM were randomly distributed into two groups. One group received 20 mg of simvastatin plus 145 mg of fenofibrate, and the other group received 20 mg of simvastatin plus 2 g of niacin plus laropiprant for a 12-week period. After a new 6-week lipid-lowering drug wash-out period, they were shifted to the other lipid-lowering drug in a crossover design for a 12-week period. 

The Hospital Ethics Committee approved the study, and all patients provided their written consent to participate in the study.

### 2.2. Clinical and Laboratory Determinations

A blood sample was obtained from each patient in the study cohort after overnight fasting. Aliquots were prepared for immediate storage at −80 °C in the BioBanc at our center prior to use. Biochemical parameters, lipids, and apolipoproteins were measured using colorimetric, enzymatic and immunoturbidimetric assays, respectively (Spinreact, SA, Spain; Wako Chemicals GmbH, Germany; and Polymedco, New York, NY, USA; CV < 4%), which were adapted to the Cobas Mira Plus Autoanalyser (Roche Diagnostics, Spain). The lipid profile was analyzed according to the Spintrol “H” CAL (Spinreact, SA, Spain) GC–MS reference methods. Spintrol “H” Normal was used as a quality control. The circulating PCSK9 levels were measured by an enzyme-linked immunosorbent assay (ELISA) kit (R&D Systems, Minneapolis, MN, USA). The FABP5, FABP4 and HMW-adiponectin levels were assessed using commercial ELISA kits (BioVendor Laboratory Medicine Inc., Brno, Czech Republic; RayBiotech, Inc., Georgia, GA, USA; CV, 5%). The serum GRP78/BiP levels were measured with an ELISA kit (Enzo Life Sciences, Inc., New York, NY, USA) following the reagent manufacturer’s instructions. The optical density (OD) of the well was measured at a wavelength of 450 nm ± 2 nm (Synergy, BioTek Instruments, Inc., Winooski, VT, USA). Each sample was analyzed in duplicate. The serum GRP78/BiP levels were measured using a standard curve constructed with the kit’s standards. The homeostasis model assessment–insulin resistance (HOMA–IR) index was calculated from the fasting glucose and insulin concentrations, as previously reported [25].

### 2.3. Carotid Ultrasound Imaging

A total of 316 subjects from the entire cohort underwent a vascular study with the Mylab 50 X-Vision ultrasound (Esaote, Italy). A 7.5 MHz linear array and semiautomated software were used to measure the carotid intima–media thickness (cIMT) in the far wall of both common carotid arteries. The cIMT mean was the average of 2 territories. Bifurcations and internal carotids were also measured using a manual method. A plaque was defined as a focal structure that either encroached into the arterial lumen by at least 0.5 mm or 50% of the surrounding cIMT value or demonstrated a thickness >1.5 mm, as measured from the media–adventitia interface to the intima-lumen interface, according to the Mannheim carotid intima–media thickness consensus [26].

### 2.4. Statistical Analyses

Data are presented as medians and 25th and 75th percentiles or percentages, unless otherwise indicated. The normality of continuous variables was determined by the Kolmogorov–Smirnov test. GRP78/BiP was log-transformed to reduce skewness. Unadjusted associations between GRP78/BiP and continuous variables were assessed by Spearman’s correlation test. Differences in GRP78/BiP between patients with obesity, DM, and MS were analyzed by the Mann–Whitney test, and differences between MS components were analyzed using the Kruskal–Wallis test. Group differences between treatments in the validation cohort were analyzed with the paired Wilcoxon test. Adjusted differences were investigated using analysis of covariance (ANCOVA). Multivariate linear regression models were constructed to search for independent relationships between GRP78/BiP (dependent variable) and clinical and biochemical variables in the whole study group and in the obese, type 2 diabetic, and MS subjects. Logistic binary regression models were also performed for dichotomous variables to assess the risk of obesity, DM, MS, or atherosclerotic plaques based on the serum GRP78/BiP levels. All statistical analyses were conducted with SPSS 25 (IBM, Armonk, NY, USA). A *p* value < 0.05 was considered statistically significant.

## 3. Results

### 3.1. Subjects’ Characterisctics

The clinical, anthropometric, and biochemical characteristics of the patients participating in the cross-sectional study are shown in Table 1. The median (25th percentile–75th percentile) age of the study subjects was 60 (50–67) years, and 50.9% were women. Obesity was present in 52.5%, DM in 72.8%, and MS in 78.6% of the patients. Carotid atherosclerotic plaques were present in 33.2% of the patients. The median GRP/BiP level was 7.43 (4.42–13.49) µg/mL. Analysis of our full cohort revealed no differences in GRP78/BiP between genders, and the level was unrelated to age. 

### 3.2. Association of GRP78/BiP with the Metabolic Status

**Figure 1:**
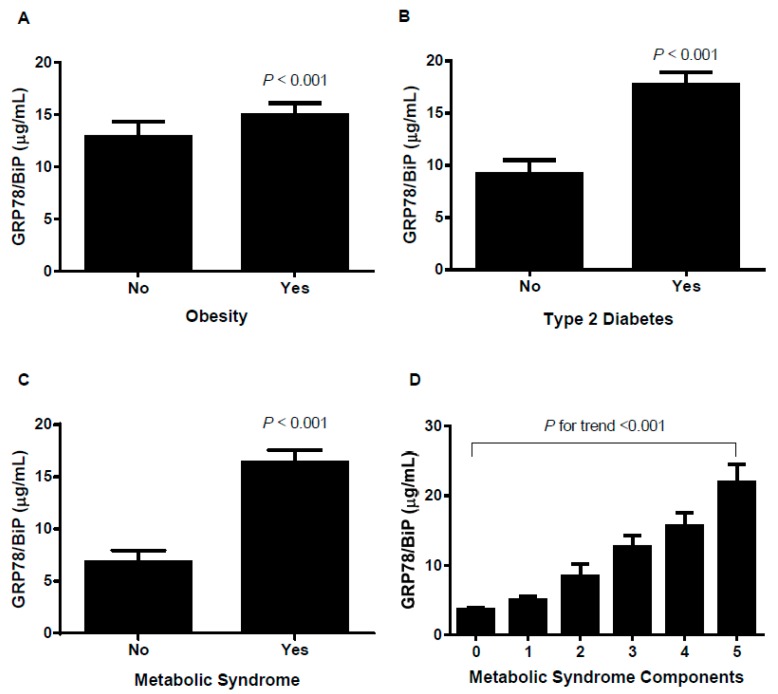
Circulating 78 kDa glucose-regulated protein/binding immunoglobulin protein (GRP78/BiP) levels according to obesity (**A**), type 2 diabetes (**B**), metabolic syndrome (**C**) and metabolic syndrome components (**D**). The results are expressed as the mean ± SEM. *p* values for group comparisons are reported for the age- and gender-adjusted ANCOVA test.

The GRP78/BiP serum concentrations were higher in patients with obesity, DM, and MS compared with patients without metabolic disturbances (5.67 (3.74–11.62) µg/mL vs. 9.15 (5.74–16.38) µg/mL, *p* < 0.001; 4.72 (3.63–9.94) µg/mL vs. 8.57 (5.27–16.73) µg/mL, *p* < 0.001 and 4.15 (2.91–5.76) µg/mL vs. 9.15 (5.85–16.73) µg/mL, *p* < 0.001, respectively) (Figure 1A,B,C). A direct and positive association was found with the number of MS components (*p* < 0.001) (Figure 1D). The differences were independent of covariates (*p* < 0.001 for obesity, DM, and MS) (Figure 1).

A logistic regression analysis revealed that the serum GRP78/BiP levels were associated with the presence of obesity, DM, and MS in the crude and in the gender- and age-adjusted model (model 1). After adjusting for other risk factors (model 2), GRP78/BiP levels remained directly associated with DM and MS (Supplemental Table S1).

### 3.3. Associations of GRP78/BiP with the Clinical, Biochemical and Vascular Imaging Data

The associations among GRP78/BiP and the clinical, anthropometric, and standard biochemical data and the adipokine and vascular values are shown in Table 2. Notably, GRP78/BiP was directly related to all adiposity indexes, including the BMI, standard lipids, lipoprotein levels, glucose concentrations, inflammation and adipokines, and was inversely related to apo A1 and HMW-adiponectin. The mean GRP78/BiP was also directly related to cIMT.

After adjusting for age, gender, and BMI, the relationships that remained statistically significant with GRP78/BiP were cholesterol, non-HDL-C, triglycerides, apoB100 and cIMT (*p* < 0.05 for all comparisons) (Table 2).

Thereafter, a multivariate stepwise regression analysis was used to identify factors influencing circulating GRP78/BiP across obesity, DM, and MS. In the entire study population, gender, systolic blood pression, triglycerides, and cIMT were the determinants of circulating GRP78/BiP, accounting for 32.7% of the observed variance (Supplemental Table S2). Further analysis of obese individuals revealed that gender accounted for 15.4% of the variance observed in the circulating GRP78/BiP levels. In diabetic individuals, gender, BMI, triglycerides, and cIMT accounted for 25.3% of the variance. For MS, the determinants of circulating GRP78/BiP were gender, triglycerides, and cIMT, which accounted for 20.3% of the variance. 

### 3.4. Associations Between GRP68/BiP and the Carotid Plaque Burden

**Figure 2:**
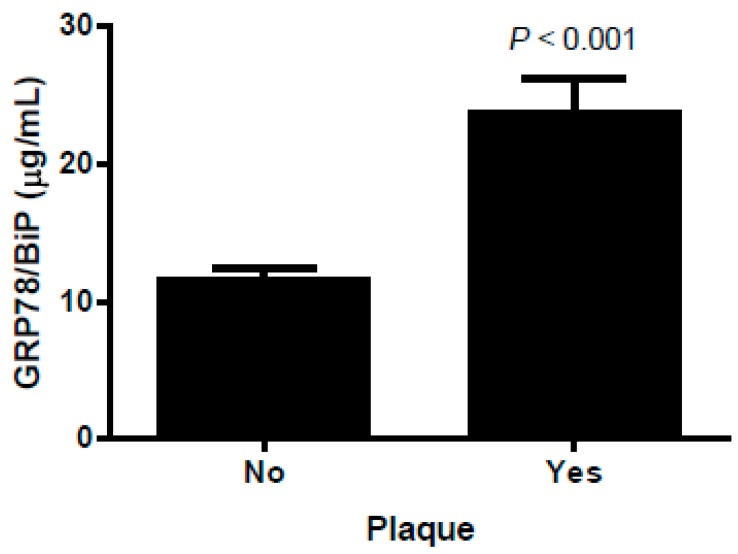
Circulating 78 kDa glucose-regulated protein/binding immunoglobulin protein (GRP78/BiP) levels according to the presence of atherosclerotic plaques. The results are expressed as the mean ± SEM. *p* values for group comparison are reported for the age- and gender-adjusted ANCOVA test.

Age- and gender-adjusted GRP78/BiP was higher in patients with carotid plaques (7.13 (4.56–12.63) µg/mL vs 11.8 (7.91–24.99) µg/mL, *p* < 0.001, *n* = 316, respectively) (Figure 2). GRP78/BiP was directly related to the carotid plaque presence (odds ratio OR, 95% confidence interval [CI] = 7.077 (3.357–14.922), *p* < 0.001) in the age- and gender-adjusted model. The direct association of GRP78/BiP with the presence of a plaque was significant in the patients with obesity (OR [CI] = 5.053 (1.716–14.876), *p* = 0.003), DM (OR [CI] = 6.296 (2.521–15.724), *p* < 0.001), and MS (OR [CI] = 5.109 (2.216–11.778), *p* < 0.001) (Supplemental Table S3). 

### 3.5. GRP68/BiP and Prediabetes 

GRP78/ BiP was increased in the subjects with prediabetes compared with the levels in the subjects with neither prediabetes nor diabetes (4.46 (3.22–8.66) µg/mL vs. 7.23 (4.54–13.96) µg/mL, *p* < 0.001, respectively). Additionally, a positive association was detected with the HOMA-IR (*r* = 0.407; *p* < 0.001, *n =* 168) that persisted after age and gender adjustment (*r* = 0.231; *p =* 0.003). In the patients with triglycerides < 2.26 mmol/L, GRP78/BiP was significantly higher in those with prediabetes and diabetes than in the controls, whereas the levels in the patients with high triglycerides (>2.26 mmol/L) were equally altered (Supplemental Figure S1). 

### 3.6. Effect of Treatment on Circulating GRP78/BiP in DM Patients

**Figure 3:**
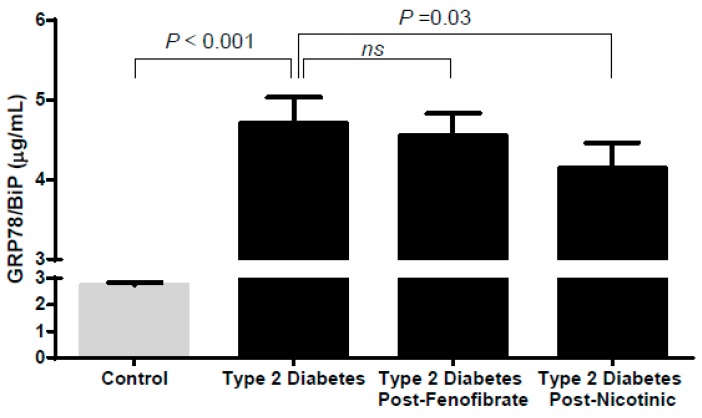
Circulating 78 kDa glucose-regulated protein/binding immunoglobulin protein (GRP78/BiP) in the control group and the type 2 diabetes patients before and after fenofibrate and nicotinic treatment in a validation cohort. The results are expressed as the mean ± SEM. *p* values for the group comparisons are reported for the age- and gender-adjusted ANCOVA test or paired Wilcoxon test.

The clinical characteristics of the patients participating in the intervention trial were previously published [24]. The median (25th percentile–75th percentiles) age was 58 (53–65) years, and 36.7% were women. Similar to the main study cohort, the age- and gender-matched GRP78/BiP levels were higher in the DM patients than in the control group (*p* < 0.001). Similar to those of the main study cohort, GRP78/BiP showed a direct association with triglycerides (*r* = 0.581; *p* < 0.001) and the cIMT (*r* = 0.509; *p =* 0.016, *n* = 22). Interestingly, in the DM patients, treatment with nicotinic acid for a 12-week period (*n* = 26) significantly reduced GRP78/BiP by 11% (*p =* 0.038) (Figure 3). This reduction was accompanied by the expected reduction in triglycerides (39%, *p =* 0.003). Treatment with fenofibrate (*n* = 29) also accounted for a significant reduction of triglycerides (32%, *p =* 0.002) but resulted in a non-significant reduction of GRP78/BiP (*p* = 0.705). 

## 4. Discussion

We communicate that the circulating GRP78/BiP levels are significantly increased in obese, DM, and MS patients. Higher GRP78/BiP concentrations are also associated with subclinical atherosclerosis. The data are robust enough to support the use of GRP78/BiP serum concentrations as a biomarker of metabolic and vascular derangements. The endoplasmic reticulum is a crucial subcellular organelle that is responsible for protein, lipid, glucose, and calcium metabolism. Conditions in which its physiological capacity is overwhelmed are referred to as ERS, in which a complex molecular reaction is activated and proteins are not properly processed due to the UPR [27]. GRP78/BiP is a cornerstone protein of this process [1]. It is physiologically located in the inner layer of the ER and maintains the localization of several proteins associated with the ERS response. In ERS situations, GRP78/BiP frees these proteins to counterbalance the UPR by reducing protein synthesis, increasing misfolded protein removal and improving the protein folding capacity. Increased extracellular delivery of GRP78/BiP occurs because of this process. According to the results of our and other studies [4], this increased secretion leads to higher circulating GRP78/BiP concentrations in humans. Thus, a high serum GRP78/BiP level should be interpreted as an ERS marker. Triglycerides and cholesterol esters are assembled in the ER. ER homeostasis is altered in the presence of a high amount of lipids, leading to ERS [28,29,30]. Therefore, our data showing an increased amount of a circulating protein associated with ERS in subjects with important alterations in intermediate metabolism are logical. According to our data, GRP78/BiP can be detected in the sera at very low levels, which increase in the presence of obesity, DM, and MS. Interestingly, a robust direct association exists between GRP78/BiP and cholesterol and triglycerides, suggesting that alterations in lipid metabolism are involved in ERS and the increase in circulating GRP78/BiP. This finding could be of interest, given that GRP78/BiP expression in vitro is associated with an increase in expression of the very low-density lipoprotein receptor [6], which is important in tissues with active fatty acid metabolism. In other words, the high fat pools in tissues involved in lipid metabolism, such as the liver and adipose tissue, would be at least partially responsible for ERS. This possibility is of interest in patients with normal triglyceride levels but signs of prediabetes or resistance to insulin; we observed that GRP78/BiP was already high in these patients, suggesting that other metabolic alterations might play a role in GRP78/BiP secretion.

On the other hand, inflammation is also associated with ERS [31]. This fact could also explain ERS in diabetes and obesity because they are associated to chronic subclinical inflammation. In our hands, GRP78/BiP was also correlated with the hsCRP concentration. 

Interestingly, triglyceride-lowering drugs have different impacts on the GRP78/BiP plasma concentration, although, as expected, both drugs produced a significant triglyceride-lowering effect. Niacin but not fenofibrate induced a significant reduction of GRP78/BiP. The lipid-lowering effect of niacin is not completely understood but seems to be mediated by a decrease in adipose tissue lipolysis, which reduces the substrate for triglyceride synthesis in the liver, whereas fenofibrate acts mainly during the catabolic phase [32,33]. The decrease in the intracellular lipid burden mediated by niacin can most likely explain the observed differences.

An interesting observation of our study is the significant association between GRP78/BiP and the presence of subclinical atherosclerosis, particularly in those with carotid plaques. We have no elements to link a direct impact of GRP78/BiP with atherosclerotic pathogenesis, although the protein is expressed in macrophages, smooth muscle cells, and endothelial cells of atherosclerotic lesions in animal models [20]. We cannot exclude the possibility that high circulating GRP78/BiP levels are signaling individuals with more severe metabolic alterations, although the correlation between GRP78/BiP and carotid plaques is maintained after multiple adjustments.

Our work has some limitations. The cross-sectional design precludes obtaining causal relationships between GRP78/BiP and metabolic and vascular alterations. The prospective, randomized and controlled validation study was open, the sample size was small, and the study was not designed for this objective. However, the data obtained are in concordance with the results from the main part of the study and provide additional information on the reversibility of ERS. 

In conclusion, the circulating GRP78/BiP levels are significantly increased in people with DM, obesity, and its associated metabolic alterations. The associated hyperlipidemia probably plays a role in ERS in these patients. GRP78/BiP was also associated with subclinical atherosclerosis. Taking all of these results together, our work supports the use of the circulating GRP78/BiP level as a marker of vascular and metabolic risk. Our data provide elements to bring derangement of a crucial cellular mechanism to the clinical setting. 

## Figures and Tables

**Table 1:** Clinical and biochemical characteristics of the study subjects.

	*N* = 405
Women (%)	50.9
Age (years)	60 (50–67)
BMI (kg/m^2^)	27.53 (27.33–34.75)
Waist circumference (cm)	103.0 (95.0–112.0)
Systolic BP (mmHg)	133 (124–146)
Diastolic BP (mmHg)	80 (71–85)
Glucose (mg/dL)	126.7 (101.0–163.0)
Insulin (%) *	10.43 (6.95–16.48)
HbA1c (%) ^†^	6.40 (5.70–7.50)
HOMA-IR *	3.13 (1.75–6.19)
**Lipids and Apolipoproteins**	
Total cholesterol (mmol/L)	5.20 (4.50–6.19)
LDL-C (mmol/L)	3.18 (2.55–3.97)
HDL-C (mmol/L)	1.38 (1.19–1.59)
Non-HDL-C (mmol/L)	3.80 (3.14–4.73)
Total triglycerides (mmol/L)	1.65 (1.04–2.58)
ApoB100 (mg/dL)	103 (85–120)
ApoA1 (mg/dL)	136 (128–146)
Lp(a) (mg/dL)	8.10 (2.70–22.00)
**Protein Biomarkers**	
GRP78/BiP (µg/mL)	7.43 (4.42–13.49)
PCSK9 (ng/mL)	320.2 (254.4–404.7)
hsCRP (mg/L)	2.09 (1.12–3.75)
FABP4 (ng/mL)	26.06 (16.84–37.24)
FABP5 (ng/mL)	7.74 (6.13–9.92)
HMW-Adiponectin (µg/mL)	5.44 (2.96–8.98)
**Disease**	
Obesity (%)	52.5
Type 2 diabetes (%)	72.8
Metabolic syndrome (%)	78.6
**Subclinical Atherosclerosis**	
cIMT (mm) ^‡^	0.685 (0.619–0.776)
Carotid atherosclerotic plaque (%) ^§^	33.2

Data are shown as n (percentage) or median (25th percentile–75th percentile). BMI = body mass index; BP = blood pressure; LDL-C = LDL cholesterol; HDL-C = HDL cholesterol; Non-HDL-C = non-HDL cholesterol; ApoB100 = apolipoprotein B100; ApoA1 = apolipoprotein A1, Lp(a) = lipoprotein a; GRP78/BiP = 78 kDa glucose-regulated protein/binding immunoglobulin protein; PCSK9 = proprotein convertase subtilisin/kexin type 9; hsCRP = high-sensitivity C-reactive protein; FABP4 = fatty acid binding protein 4; FABP5 = fatty acid binding protein 5; HMW-adiponectin = high-molecular-weight adiponectin; cIMT = carotid intima–media thickness. Measurements were available in a subpopulation of: * *n* = 168; ^†^ *n* = 314; ^‡^ *n* = 312; ^§^ *n* = 316.

**Table 2:** Relationships between log-GRP78/BiP and continuous variables.

	Log GRP78/BiP	*p Value*	Log GRP78/BiP *	*p Value* *
Age	0.034	0.499	-	-
BMI	0.307	<0.001	-	-
Waist Circumference	0.269	<0.001	0.125	0.121
Systolic BP	0.394	<0.001	0.154	0.055
Diastolic BP	0.153	<0.001	0.122	0.129
Glucose	0.296	<0.001	−0.045	0.576
Total cholesterol	0.429	<0.001	0.156	0.052
LDL-C	0.351	<0.001	0.099	0.217
HDL-C	−0.011	0.830	0.038	0.637
Non-HDL-C	0.472	<0.001	0.176	0.028
Total triglycerides	0.392	<0.001	0.243	0.002
ApoB100	0.420	<0.001	0.169	0.035
ApoA1	−0.165	0.001	−0.137	0.088
Lp(a)	0.065	0.190	0.039	0.626
PCSK9	0.191	<0.001	0.077	0.340
hsCRP	0.256	<0.001	0.084	0.297
FABP4	0.141	0.005	0.104	0.195
FABP5	0.274	<0.001	0.095	0.236
HMW-Adiponectin	−0.176	0.001	−0.055	0.493
cIMT	0.165	0.003	0.244	0.002

GRP78/BiP = 78 kDa glucose-regulated protein/binding immunoglobulin protein; BMI = body mass index; BP = blood pressure; LDL-C = LDL cholesterol; HDL-C = HDL cholesterol; Non-HDL-C = non-HDL cholesterol; ApoB100 = apolipoprotein B100; ApoA1 = apolipoprotein A1, Lp(a) = lipoprotein a; PCSK9 = proprotein convertase subtilisin/kexin type 9; hsCRP = high-sensitivity C-reactive protein; FABP4 = fatty acid binding protein 4; FABP5 = fatty acid binding protein 5; HMW-adiponectin = high-molecular-weight adiponectin; cIMT = carotid intima–media thickness. *p* values for Spearman’s correlations are reported. * *p* values corrected by age, gender and BMI.

## Supplementary Materials

The following are available online at https://www.mdpi.com/2077-0383/8/11/1793/s1.
