# Supplementary material for: The Circulating GRP78/BiP Is a Marker of Metabolic Diseases and Atherosclerosis: Bringing Endoplasmic Reticulum Stress into the Clinical Scenario
Source: J Clin Med. 2019 Oct 26;8(11):1793. doi: 10.3390/jcm8111793 (PMC6912434; doi:10.3390/jcm8111793)
Supplement: Supplementary file 1 [file jcm-08-01793-s001.pdf]

Supplemental Material

Supplemental Table S1: Crude and adjusted models used to assess the association between the log-GRP78/BiP and metabolic status.

Group	Obesity		Type 2 Diabetes		Metabolic Syndrome	
	**OR (95% CI)**	***p***	OR (95% CI)	*P*	OR (95% CI)	*P*
Crude model	2.86 (1.70–4.82)	<0.001	4.24(2.24–8.00)	<0.001	19.13 (8.05–45.47)	<0.001
Adjusted model 1	2.86 (1.69–4.80)	<0.001	4.46 (2.26–8.79)	<0.001	18.66 (7.85–44.36)	<0.001
Adjusted model 2	1.56 (0.85–2.80)	0.157	2.26 (1.07–4.76)	0.033	4.06 (1.45–11.31)	0.007

Logistic regression models (odds ratio; OR and 95% confidence interval; CI). Model 1 was adjusted by age and gender, and model 2 was adjusted by age, gender, BMI (not included in obesity), glucose (not included in type 2 diabetes), total cholesterol, apoB100, Apo A1, total triglycerides, and systolic BP. GRP78/BiP indicates 78 kDa glucose-regulated protein/binding immunoglobulin protein, BMI, body mass index; ApoB100, apolipoprotein B100; ApoA1, apolipoprotein A1; BP, blood pressure.

Supplemental Table S2. Variables that were significantly associated with log-GRP78/BiP in the multivariate stepwise regression analysis.

Independent variables	Beta	*p*	R^2
**All**			0.327
Gender	−0.197	0.002	
Systolic BP	0.226	0.004	
Total triglycerides	0.250	0.001	
cIMT	0.162	0.002	
**Obesity**			0.154
Gender	−0.220	0.049	
**Type 2 diabetes**			0.253
Gender	−0.209	<0.001	
BMI	−0.019	0.023	
Total triglycerides	0.001	0.025	
cIMT	0.395	0.001	
**Metabolic Syndrome**			0.203
Gender	−0.193	0.020	
Total triglycerides	0.269	0.005	
cIMT	0.200	0.002	

Linear regression models for the log-GRP78/BiP dependent variable (standardized estimates, *p*-value reported and R^2 for the adjusted model). The variables included were age, gender, BMI, waist circumference, glucose, total cholesterol, systolic BP, diastolic BP, total triglycerides, non-HDL-C, ApoB100, ApoA1 and cIMT. GRP78/BiP indicates 78 kDa glucose-regulated protein/binding immunoglobulin protein; BMI, body mass index; BP, blood pressure; non-HDL-C, non-HDL cholesterol; ApoB100, apolipoprotein B100; ApoA1, apolipoprotein A1; cIMT, carotid intima-media thickness.

Supplemental Table S3. Association between log-GRP78/BiP and atherosclerotic plaque in a subgroup of 316 patients and according to the cardiometabolic status.

	**OR (95% CI)**	***p***
All	7.212(3.228–16.113)	<0.001
Obesity	4.167(1.407–12.334)	0.010
Type 2 Diabetes	7.500(2.634–21.354)	<0.001
Metabolic Syndrome	5.004(2.083–12.024)	<0.001

Logistic regression models (odds ratio; OR and 95% confidence interval; CI) were adjusted by gender, BMI, total triglycerides and systolic BP. GRP78/BiP indicates 78 kDa glucose-regulated protein/binding immunoglobulin protein; BMI; body mass index and BP, blood pressure.

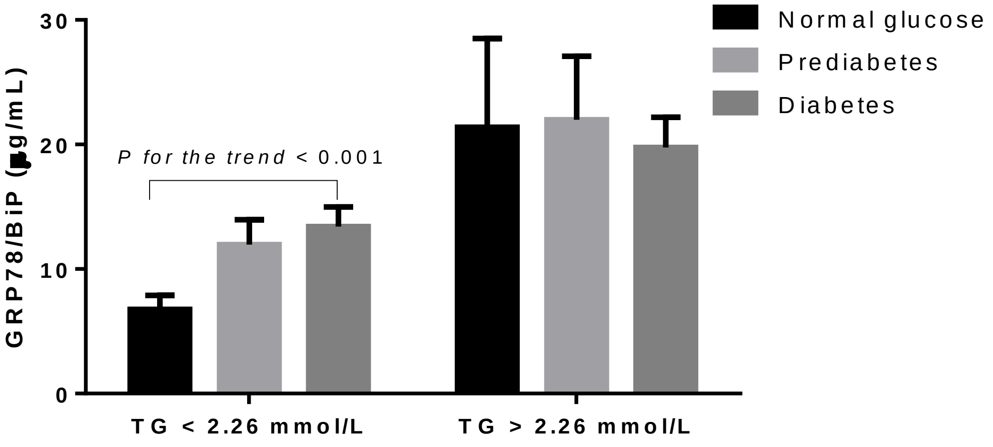

Supplemental Figure S1. Mean values and their SEMs of the circulating BiP/GRP78 levels across the fasting glucose categories (normal glucose < 100 mg/dL; prediabetes 100–125 mg/dL and type 2 diabetes >126 mg/dL) and triglycerides (TG < 2.26 and TG > 2.26 mmol/L). The *p* values (from the ANCOVA test adjusted for age and gender) indicate statistical significance. GRP78/BiP indicates 78 kDa glucose-regulated protein/binding immunoglobulin protein and TG, total triglycerides.
